# Analiza Przydatności Pomiarów Tlenku Azotu w Powietrzu Wydychanym w Diagnostyce Oraz Leczeniu Alergicznego Nieżytu Nosa i Astmy u Dzieci i młodzieży

**DOI:** 10.34763/devperiodmed.20182202.135143

**Published:** 2018-06-30

**Authors:** Agnieszka Kowalczyk, Aneta Krogulska

**Affiliations:** 1 Katedra i Klinika Pediatrii, Alergologii i Gastroenterologii Collegium Medicum im. Ludwika Rydygiera w Bydgoszczy Uniwersytetu Mikołaja Kopernika w Toruniu, Polska

**Keywords:** tlenek azotu, FeNO, NOS, astma, alergiczny nieżyt nosa, nNO, nitric oxide, FeNO, NOS, asthma, allergic rhinitis, nNO

## Abstract

Tlenek azotu, powstający przy udziale enzymów z grupy syntaz tlenku azotu, spełnia w organizmie człowieka szereg istotnych funkcji, ale produkowany w nadmiarze wykazuje działanie prozapalne. Pomiary stężenia tlenku azotu w powietrzu wydychanym znajdują zastosowanie w diagnostyce i monitorowaniu nasilenia stanu zapalnego dróg oddechowych o podłożu eozynofilowym, nie powinny być jednak wykorzystywane jako samodzielny parametr w diagnozowaniu astmy lub monitorowaniu jej leczenia. Ocena stężeń tlenku azotu w powietrzu wydychanym znajduje również zastosowanie w ustalaniu patogenezy objawów u pacjentów z nieżytem nosa. Jest także pomocna w wykrywaniu i monitorowaniu współistniejącego eozynofilowego stanu zapalnego w dolnych drogach oddechowych u tych chorych. Wartości tlenku azotu w powietrzu wydychanym mogą być nieprawidłowe (zarówno obniżone, jak i podwyższone) w innych chorobach przewlekłych takich jak: mukowiscydoza, pierwotna dyskineza rzęsek oraz nieswoiste zapalenia jelit. Na wartości pomiaru wpływają liczne czynniki, takie jak: atopia, polimorfizmy genów syntaz tlenku azotu i parametry przemiany lipidowej organizmu. Oprócz pomiarów tlenku azotu w powietrzu wydychanym przydatne są także pomiary nosowego tlenku azotu, które znalazły zastosowanie w ocenie występowania oraz nasilenia zapalenia eozynofilowego w górnych drogach oddechowych.

## Abstract (en)

Nitric oxide is produced by enzymes called nitric oxide synthases. It fulfills many important functions in the human body, but produced in excess amount has a proinflammatory activity. Fractional exhaled nitric oxide measurements are used in the diagnosis and monitoring of eosinophilic inflammation in the lower airways, but should not be used as an independent parameter to make a diagnosis of asthma or for the monitoring of asthma treatment. Evaluation of fractional exhaled nitric oxide concentrations is also used to determine the pathogenesis of symptoms in patients with rhinitis. In addition, they are helpful in detecting and monitoring eosinophilic inflammation in the lower respiratory tract that coexists with inflammation in the upper airways. Fractional exhaled nitric oxide concentrations may be abnormal (lowered or elevated) in other chronic diseases, such as cystic fibrosis, primary ciliary dyskinesia and inflammatory bowel diseases. Many factors, e.g. atopy, genetic polymorphisms of NOS, and the lipid profile affect the fractional exhaled nitric oxide measurement. Nasal nitric oxide measurement is useful in assessing the prevalence and severity of eosinophilic inflammation in the upper respiratory tract.

## Znaczenie tlenku azotu w organizmie człowieka

Tlenek azotu (NO − nitric oxide) jest produkowany w organizmie człowieka z L-argininy w reakcji katalizowanej przez enzymy z grupy syntaz tlenku azotu (NOS − nitric oxide synthase). Gaz ten pełni szereg fizjologicznych funkcji, m.in. działa relaksująco na mięśniówkę gładką naczyń krwionośnych, hamuje agregację płytek krwi, bierze udział w przewodnictwie nerwowym oraz procesach immunologicznych [1, 2]. Niewielkie ilości NO, niezbędne do prawidłowego funkcjonowania organizmu są wytwarzane przez dwie konstytutywne izoformy enzymu − neuronalną (nNOS − neuronal nitric oxide synthase, NOS I) oraz śródbłonkową (eNOS − endothelial nitric oxide synthase, NOS III) [3, 4]. Ekspresję trzeciej izoformy NOS − indukowalnej (iNOS − inducible nitric oxide synthase, NOS II) wykazano m.in. na komórkach nacieku zapalnego. Kontakt z alergenem, na który pacjent jest uczulony, zapoczątkowuje reakcję alergiczną i wydzielanie cytokin prozapalnych (IL-1, IFN gamma, TNF alfa) pobudzających ekspresję iNOS. Prowadzi to do długotrwałego wydzielania dużych ilości NO [5, 6]. Na komórkach nabłonka oskrzeli stwierdzono obecność wszystkich trzech izoform NOS [3]. W błonie śluzowej nosa u osób zdrowych wykazano ekspresję form konstytutywnych syntazy, natomiast w śluzówce zatok przynosowych głównie izoformy iNOS [4]. Tlenek azotu w niewielkich stężeniach wywiera pozytywny wpływ na funkcjonowanie układu oddechowego, m.in. wykazuje działanie relaksujące na mięśniówkę gładką ściany oskrzeli [7, 8, 9]. Z kolei duże ilości NO działają prozapalnie i sprzyjają rozwojowi nadreaktywności oskrzeli [1, 8, 10]. W górnych drogach oddechowych NO wpływa na ruchomość rzęsek błony śluzowej i pomaga w prawidłowym oczyszczaniu zatok [11].

## Pomiary FeNO u pacjentów z astmą

FeNO w diagnostyce astmy

ATS (Amerykańskie Towarzystwo Klatki Piersiowej) zaleca wykonywanie pomiarów stężenia NO w powietrzu wydechowym (f*ractional* exhaled *nitric oxide* − *FeNO)* celem:

− diagnostyki stanów zapalnych dróg oddechowych o podłożu eozynofilowym,
− oceny prawdopodobieństwa odpowiedzi na sterydoterapię w grupie pacjentów z przewlekłymi objawami ze strony układu oddechowego, jeśli podejrzewamy że dolegliwości mają podłoże zapalne,
− monitorowaniu nasilenia stanu zapalnego w drogach oddechowych u pacjentów chorujących na astmę.

Należy podkreślić, że pomiary FeNO jako samodzielny parametr nie powinny być stosowane w diagnostyce astmy, w której podstawowe znaczenie mają kryteria kliniczne [12]. Najnowsze wytyczne GINA 2017 również wskazują, że wartości FeNO nie są użyteczne do potwierdzenia ani wykluczenia w toku diagnostyki astmy [13]. ATS zwraca jednak uwagę na przydatność pomiarów FeNO jako obiektywnego dowodu na obecność zapalenia eozynofilowego w drogach oddechowych w trakcie diagnostyki astmy. Należy przy tym pamiętać, że w przypadku stanu zapalnego o podłożu nieeozynofilowym wartości FeNO są zazwyczaj prawidłowe [12]. Wykazano, że w grupie dzieci do 4r.ż, wysokie wartości FeNO wraz z występowaniem nawracającego kaszlu i świstów, niezwiązanych z infekcją, mogą poprzedzać diagnozę astmy w wieku szkolnym [14]. Interpretację wartości FeNO opartą na zaleceniach ATS oraz najnowszych wynikach badań klinicznych przedstawia Tabela I [15]. Zatem obecnie pomiary FeNO są jedną ze składowych procesu diagnostycznego u pacjentów z objawami chorobowymi dotyczącymi dolnych dróg oddechowych i mogą wskazywać na istnienie stanu zapalnego o podłożu eozynofilowym. Należy podkreślić, że niezmiernie ważne jest by interpretować wyniki badania FeNO w ścisłej korelacji z obrazem klinicznym.

**Tabela I:** Interpretacja pomiarów FeNO u pacjentów z podejrzeniem lub zdiagnozowaną astmą [15]. Table I. Interpretation of FeNO values in the management of patients with suspected or diagnosed asthma [15].

	Interpretacja wyniku FeNO *Interpretation of FeNO values*		
	Stężenia FeNO (ppb) *FeNO concentrations (ppb)*		
	Prawidłowe*Normal*	Podwyższone*Elevated*	Wysokie*High*
**Dorośli** ***Adults***	<20-25^*^	20/25-50	<50
**Dzieci** ***Children***	<15-20^*^	15/20-35	<35
Stan zapalny Th2-zależny *Th2-mediated inflammation*	Mało prawdopodobny *Unlikely*	Prawdopodobny *Likely*	Znaczący *Significant*
**Postępowanie z pacjentem z podejrzeniem astmy^1)^*Managing of the patient with suspected asthma^1)^***			
Znaczenie w diagnostyce astmy *Importance in the diagnosis of asthma*	Należy rozważyć inną niż astma przyczynę objawów *Other than asthma cause of the symptoms should be considered*	Diagnoza astmy jest prawdopodobna *The diagnosis of asthma is likely*	Diagnoza astmy jest prawdopodobna *The diagnosis of asthma is likely*
Znaczenie w podejmowaniu decyzji terapeutycznej *Importance in making a therapeutic decision*	Odpowiedź na sterydoterapię wziewną mało prawdopodobna *Response to inhaled corticosteroid therapy is unlikely*	Odpowiedź na sterydoterapię wziewną prawdopodobna *Response to inhaled corticosteroid therapy is likely*	Odpowiedź na sterydoterapię wziewną prawdopodobna *Response to inhaled corticosteroid therapy is likely*
**Postępowanie w trakcie leczenia pacjenta z astmą^1)^** ***Managing of the patient during asthma treatmen^1)^***			
Zalecenia dotyczące opieki nad pacjentem *Patient care*	Stan zapalny Th2-zależny jest pod kontrolą *Th2-mediated inflammation is under control*	Sprawdź *Check-*. - przestrzeganie zaleceń dotyczących leczenia, *compliance to treatment recommendations*, - technikę inhalacji, *inhaler technigue*, - możliwe narażenie na alergeny na które pacjent jest uczulony *possible exposure to allergens to which the patient is sensitized*	Sprawdź *Check*: - przestrzeganie zaleceń dotyczących leczenia, *compliance to treatment recommendations*, - technikę inhalacji, *inhaler technigue*, - możliwe narażenie na alergeny na które pacjent jest uczulony *possible exposure to allergens to which the patient is sensitized* Wskazuje na zwiększone ryzyko wystąpienia zaostrzenia astmy, niezależnie od przebiegu klinicznego choroby *Increased risk of asthma exacerbation, regardless of the clinical course of the disease*
Zalecenia dotyczące decyzji o zmianie leczenia *Making decision about changing the asthma treatment*	Jeżeli astma jest kontrolowana przez ostatnie 3-6 mc-y, rozważ zmniejszenie intensywności leczenia przeciwzapalnego *If asthma is controlled over the last 3-6 months, consider reducing the intensity of anti-inflammatory therapy*	Jeżeli wystąpiły zaostrzenia astmy, zintensyfikuj leczenie przeciwzapalne *If asthma exacerbations observed, intensify anti-inflammatory therapy*	Zintensyfikuj leczenie przeciwzapalne, szczególnie jeśli współwystępuje eozynofilia krwi lub rozważ zastosowanie drobnocząsteczkowych sterydów wziewnych *Intensify anti-inflammatory therapy, especially if blood eosinophilia is present or consider the use of small molecule inhaled steroids* Wynik może sugerować astmę sterydooporną i potrzebę włączenia systemowego leczenia przeciwzapalnego *The result may suggest steroid-resistant asthma and the necessity to include systemic anti-inflammatorytherapy of asthma*
^*^zależnie od wieku, wzrostu i płci *depending on age, height and sex*			

1) Obecne wytyczne ATS oraz GINA 2017 podkreślają, że zarówno rozpoznanie, jak i leczenie astmy nie może być oparte jedynie na wartościach FeNO. Pomiary FeNO mają charakter pomocniczy w opiece nad pacjentem z podejrzeniem i diagnozą astmy. The current ATS and GINA 2017 guidelines underline that both the diagnosis and treatment of asthma cannot be based solely on FeNO values. FeNO measurements have an auxiliary role in the care of the patient with suspected and diagnosed asthma.

### FeNO w leczeniu astmy

Reakcja alergiczna w przebiegu astmy jest mediowana przez komórki tuczne i limfocyty Th2. Wydzielane w jej trakcie cytokiny, w tym interleukiny IL-4 i IL-13, mają zdolność pobudzania ekspresji iNOS [16]. Sterydy oraz antagoniści receptora leukotrienowego (LTRA − Leukotrine receptor antagonists) hamując uwalnianie obu tych interleukin, powodują spadek wytwarzania NO w drogach oddechowych, a co za tym idzie obniżenie wartości FeNO [17]. Hanania i wsp. sugerują, że pomiary FeNO mogą być przydatne w przewidywaniu odpowiedzi na omalizumab. Wyższe wartości FeNO u pacjentów z ciężką, niekontrolowaną astmą, prognozują lepszą odpowiedź na leczenie omalizumabem niż w przypadku pacjentów z niskimi stężeniami FeNO przed włączeniem leku [18]. By dokładniej określić rolę FeNO podczas tej terapii niezbędne są jednak dalsze badania. Neelamegan i wsp. sugerują, że pomiary FeNO mogą być przydatne do monitorowania stanu zapalnego w drogach oddechowych u pacjentów chorujących na astmę, natomiast nie pozwalają na ocenę stopnia jej ciężkości i prognozowania odpowiedzi na sterydoterapię (beklometazon) [19].

Wg wytycznych ATS za znaczący z punktu widzenia klinicznego uważa się wzrost wartości FeNO u danego pacjenta pomiędzy kolejnymi pomiarami o:

− więcej niż 20% dla wartości początkowych >50 ppb lub
− więcej niż 10 ppb w przypadku wartości początkowych <50 ppb.

Z kolei za znaczącą odpowiedź na leczenie sterydami uważa się zmniejszenie wartości kontrolnego FeNO o:

− co najmniej 20% gdy wartości wynosiły >50 ppb lub
− więcej niż 10 ppb dla wartości <50 ppb.

Najnowsze wytyczne GINA 2017 nie rekomendują monitorowania leczenia astmy na podstawie oceny stężenia FeNO. Korelację pomiędzy wartościami FeNO i krótkotrwałą, dobrą odpowiedzią na sterydoterapię zaobserwowano jedynie w przypadku osób dorosłych z wartościami FeNO >50 ppb. Trudno przewidzieć jakie byłyby skutki nie zastosowania sterydów u pacjentów z niskimi wartościami FeNO, stąd w świetle obecnych badań niskie wartości FeNO nie powinny być czynnikiem decydującym o niezastosowaniu sterydoterapii u pacjentów chorujących na astmę [12, 13]. Warto ponadto zauważyć, że wysokie wartości FeNO u dorosłych chorujących na astmę alergiczną zaliczono do niezależnych czynników ryzyka zaostrzenia choroby [20].

Podsumowując, pomiary FeNO znajdują zastosowanie w monitorowaniu nasilenia stanu zapalnego w dolnych drogach oddechowych u pacjentów z astmą w trakcie jej leczenia. Jednak leczenie astmy nie powinno być rozpoczynane i prowadzone jedynie na podstawie wartości FeNO. Dalszych badań wymaga wpływ innych grup leków stosowanych u chorych na astmę, w tym omalizumabu, na mierzone wartości FeNO.

## Pomiary FeNO u pacjentów z alergicznym nieżytem nosa

W grupie dzieci chorujących na alergiczny nieżyt nosa (ANN) obserwuje się wyższe wartości pomiarów FeNO zarówno z dolnych, jak i z górnych dróg oddechowych. Stężenie FeNO koreluje z wiekiem dziecka, wzrostem oraz wartością nosowego NO (nNO − nasal nitric oxide). Natomiast dyskusyjny jest związek FeNO z objawami ANN (katar, kichanie, świąd, zatkanie nosa). Liu i wsp. nie obserwowali związku pomiędzy wartościami FeNO i nNO a objawami nieżytu nosa [21]. Z kolei Lee i wsp. wykazali istotną statystycznie zależność stężenia FeNO z nasileniem wodnistego kataru oraz świądu nosa, a także z ogólnym nasileniem objawów ANN (mierzonym jako suma poszczególnych objawów) [22]. Ziętkowski i wsp. obserwowali wyższe stężenia FeNO u dorosłych z alergicznym sezonowym nieżytem nosa (ASNN) przed okresem pylenia w porównaniu z osobami zdrowymi. Kontakt z alergenami w okresie pylenia powodował znaczący statystycznie wzrost stężenia FeNO. Na tej podstawie autorzy sugerują, że pomiary FeNO u pacjentów z ASNN mogą być wykorzystywane do monitorowania nasilenia stanu zapalnego w dolnych drogach oddechowych, współwystępującego z ANN [23]. W grupie dzieci 3-7-letnich chorujących na ANN bez współistniejącej astmy mierzone stężenia FeNO są wyższe w porównaniu zarówno ze zdrową grupą kontrolną, pacjentami z atopią bez objawów nieżytu, jak i dziećmi cierpiącymi na nieżyt nosa nieatopowy. Autorzy sugerują możliwość wykorzystania FeNO jako markera diagnostycznego w kierunku ANN w przypadku występowania objawów nieżytu nosa w tej grupie wiekowej [24]. Z kolei Ciprandi i wsp. w grupie dorosłych chorujących na ANN, zaobserwowali związek podwyższonych wartości FeNO z dłuższym okresem występowania objawów i cięższym przebiegiem ANN oraz częstszym występowaniem nadrektywności oskrzeli. W dwuletniej obserwacji, średnio co czwarty pacjent ze stężeniem FeNO >50 ppb zachorował na astmę. Stąd można wnioskować, że wysokie stężenia FeNO (>50ppb) mierzone u pacjentów z ANN mogą być czynnikiem ryzyka rozwoju astmy w przyszłości [25].

Na podstawie wyników dotychczasowych badań sugeruje się, że pomiary FeNO znajdują zastosowanie w ocenie nasilenia procesu zapalnego w dolnych drogach oddechowych u pacjentów z ANN, co może pozwolić na wyselekcjonowanie chorych szczególnie podatnych na zachorowanie na astmę w przyszłości. Dodatkowo mogą być przydatne w diagnostyce nieżytu nosa, wskazując na jego alergiczny charakter oraz wykazują związek z czasem trwania i przebiegiem ANN.

## Czynniki wpływające na pomiary FeNO

### Choroby przewlekłe i infekcje dróg oddechowych

W interpretacji wyników FeNO należy uwzględniać możliwy udział czynników mających wpływ na pomiary FeNO. Obniżone wartości FeNO obserwuje się m.in. u pacjentów z pierwotną dyskinezą rzęsek oraz mukowiscydozą, co wynika ze zmniejszonej aktywności zarówno kontytutywnych, jak i indukowalnej izoformy NOS oraz zwiększonego katabolizmu NO. Dodatkowo zwiększona ilość wydzieliny w drogach oddechowych u tych chorych powoduje zaburzenie dyfuzji NO do światła oskrzeli i w rezultacie również obniża stężenie mierzonego FeNO [26, 27, 28, 29]. Wpływ wirusowych infekcji układu oddechowego na wartości FeNO jest zależny od etiologii. W przypadku zakażeń rhinowirusem obserwowano wzrost wartości FeNO prawdopodobnie w wyniku stymulacji przez wirusa ekspresji iNOS w nabłonku dróg oddechowych. Z kolei zakażenia o etiologii RSV oraz wirusem grypy powodowały obniżenie FeNO [30, 31, 32, 33]. W przypadku dzieci urodzonych z bardzo małą masą urodzeniową, chorujących na dysplazję oskrzelowopłucną wartości FeNO mierzone w wieku szkolnym są niższe w porównaniu z rówieśnikami [34]. Również atopia jest dodatkowym czynnikiem wpływającym na wartości FeNO. Wśród dzieci chorujących na astmę, najwyższe wartości FeNO obserwowano u pacjentów uczulonych na więcej niż jeden alergen, natomiast najniższe stężenia FeNO wykazano u chorych bez atopii. Podwyższone wartości FeNO wykazano zwłaszcza w przypadku uczulenia na alergeny tzw. całoroczne (roztocza kurzu domowego, pleśnie oraz sierść zwierząt). W tej grupie pacjentów stwierdzono także pozytywny związek między wysokimi wartościami FeNO a eozynofilią w krwi obwodowej. Równocześnie wykazano, że wśród dzieci bez astmy atopia nie ma wpływu na stężenia FeNO [35]. Podwyższone wartości FeNO obserwowano także u dzieci chorujących na nieswoiste zapalenia jelit (NZJ), nieazależnie od nasilenia stanu zapalnego w jelitach [36]. Ikonomi E. i wsp. sugerują jednak, że pomiary FeNO nie są użyteczne zarówno w diagnostyce, jak i ocenie aktywności NZJ [37].

### Czynniki środowiskowe i wysiłek oddechowy

Czynnikiem mającym wpływ na pomiary FeNO, jest nikotyna. Narażenie na dym tytoniowy obniża wartości FeNO, zależnie od ilości papierosów wypalanych dziennie [38]. Z kolei dieta bogata w związki azotu podwyższa FeNO nawet o 40-60%, ze szczytem 1-2 h po spożyciu [39]. Pomiar FeNO może być zaburzony przez same manewry oddechowe. Jeśli u pacjenta planuje się wykonanie zarówno badania FeNO, jak i spirometrii, pomiar wydechowego tlenku azotu powiniem być wykonany, jako pierwszy, aby uniknąć fałszywego zaniżenia wyniku spowodowanego manewrami natężonego oddychania [11]. Zaniżone stężenie NO w powietrzu wydychanym może być także spowodowane wysiłkiem fizycznym oraz skurczem oskrzeli [40]. Jedną z głównych zalet oznaczeń FeNO w porównaniu z badaniem spirometrycznym jest prostota wykonania, co ma szczególne znaczenie w grupie młodszych dzieci, które często nie potrafią wykonać poprawnych, powtarzalnych manewrów natężonego wydechu [41, 42].

### Warianty genetyczne eNOS

Podwyższone wartości FeNO u chorych na astmę wiążą się głównie z nadeekspresją iNOS, jednak badania wskazują, że także warianty genetyczne eNOS mogą mieć znaczenie w rozwoju astmy i wpływać na ilość NO produkowanego w drogach oddechowych. Lee i wsp. wykazali częstsze występowanie polimorfizmu genu eNOS (bb) u pacjentów z astmą w porównaniu ze zdrową grupą, bez korelacji z nasileniem choroby [43]. Bouzigon i wsp. zaobserwowali u pacjentów z astmą istotną zależność pomiędzy stężeniem FeNO a polimorfizmem pojedyńczych nukleotydów (SNP - single nucleotide polymorphism) w obrębie eNOS (rs743507). Z kolei w grupie kontrolnej (bez objawów i diagnozy astmy) stężenie FeNO korelowało z dwoma SNP w obrębie eNOS (rs2853796 i rs1549758) oraz jednym SNP iNOS ( rs6505510) [44]. Warianty genetyczne eNOS mogą być związane z ryzykiem uczulenia na niektóre alergeny. Iordanidou i wsp. wykazali związek polimorfizmów w obrębie genu eNOS (-786T/C i 894G>T) z uczuleniem na sezonowe alergeny wziewne (Olea europaea, Parietaria judaica, mixed grass) u dzieci chorujących na astmę. Autorzy nie obserwowali natomiast istotnych różnic w częstości występowania badanych genotypów pomiędzy dziećmi chorujacymi na astmę nieuczulonymi na badane alergeny oraz grupą kontrolną [45].

### Parametry przemiany lipidowej i nadmierna masa ciała

Wykazano, że nieprawidłowości w zakresie przemiany lipidowej przyczyniają się do indukcji zapalenia naczyń krwionośnych i rozwoju miażdżycy [46, 47]. Zaobserwowano, że hipercholesterolemia może także ukierunkowywać odpowiedź immunologiczną w kierunku Th2, leżącej u podłoża schorzeń alergicznych [48]. Zarówno otyłość, jak i dieta o wysokiej zawartości tłuszczów są uważane za czynniki ryzyka rozwoju ANN u dzieci [49, 50]. Sugeruje się, że różne frakcje cholesterolu, w odmienny sposób wpływają na atopię i stężenie FeNO. Wyższe wartości HDL w grupie dzieci matek z astmą były związane z mniejszym ryzykiem uczulenia na alergeny wziewne oraz z niższymi wartościami FeNO. Z kolei u pacjentów z wyższymi stężeniami triglicerydów we krwi, wykazano większe ryzyko uczulenia na alergeny wziewne oraz wyższe stężenia FeNO [51].

U pacjentów otyłych, obserwuje się niższe wartości FeNO w porównaniu z rówieśnikami o prawidłowej masie ciała [52]. Yao i wsp. oceniali zależność pomiędzy nadmierną masą ciała (mierzoną za pomocą BMI - body mass index) a stężeniem FeNO u dzieci w wieku 5-18 lat. Zaobserwowali, że wśród dzieci z atopią pacjenci otyli charakteryzują się istotnie niższymi wartościami FeNO w porównaniu z dziećmi o prawidłowej masie ciała. Podobnych zależności nie zaobserwowano w grupie dzieci bez atopii [53]. Z kolei u pacjentów z już rozpoznana astmą i wyższymi wartościami FeNO wskazującymi na eozynofilowy charakter zapalenia, nieprawidłowo wysokie wartości BMI wiązały się z cięższym przebiegiem i gorszą kontrolą astmy [54].

Wydaje się, że zaburzenia przemiany lipidowej wiążą się, w nie do końca poznanym mechanizmie, z uczuleniem na alergeny wziewne oraz nasileniem eozynofilowego stanu zapalnego w obrębie dróg oddechowych, mierzonego za pomocą FeNO. Równocześnie u pacjentów otyłych z reguły obserwuje się niższe stężenia FeNO. Stąd wpływ parametrów przemiany lipidowej oraz nadmiernej masy ciała na nasilenie zapalenia eozynofilowego w dolnych drogach oddechowych jest niejasny i wymaga dalszych badań. Zagadnienia te są istotne kliniczne, ponieważ sugerują, że prawidłowa dieta i zdrowy tryb życia mogą zmniejszać ryzyko m.in. ciężkiego przebiegu astmy.

## Pomiary tlenku azotu z górnych dróg oddechowych (nNO)

Jamy nosowe, system zatok przynosowych, ucho środkowe oraz nosowa część gardła tworzą połączony ze sobą kompleks, którego każda składowa ma wpływ na wynik pomiaru nNO [55]. Większość NO w obrębie układu oddechowego jest produkowana w górnych drogach oddechowych, zwłaszcza w zatokach przynosowych. Stąd też wartości nNO są fizjologicznie wyższe niż FeNO [56]. Wg Struben i wsp. średnie wartości nNO mierzone u zdrowych dzieci w wieku 6-17 lat wynosiły 449 ppb i były niezależne od płci, wagi, wzrostu, BMI i narażenia na dym tytoniowy. Dla dzieci <12 r.ż. wykazano dodatnią korelację z wiekiem [57]. W przypadku pierwotnej dyskinezy rzęsek, obserwuje się bardzo niskie wartości nNO (<100 ppb) [58, 59]. Liczne badania wskazują na możliwość wykorzystania pomiarów nNO w diagnostyce i monitorowaniu leczenia ANN. Krzych-Fałta i wsp. przeprowadzili analizę stężeń nNO i FeNO w odpowiedzi na donosową próbę prowokacji alergenem w grupie pacjentów uczulonych na pospolite alergeny środowiskowe. We wszystkich pomiarach (przed podaniem alergenu, w 5, 10, 15 i 20 minucie oraz 4 godzinie po podaniu alergenu) zaobserwowali znacząco wyższe wartości nNO u pacjentów z ANN w porównaniu z grupą kontrolną. Mierzone wartości były wyższe (ale statystycznie nieistotnie) w grupie pacjentów z przewlekłym ANN, w porównaniu do grupy z okresowym ANN. We wczesnej fazie reakcji alergicznej zaobserwowano spadek wartości nNO w porównaniu z pomiarem przed próbą (1137,03 vs 879,53 ppb), które ponownie narosły po 4 h od prowokacji alergenem (1150,50 ppb). Początkowe zmniejszenie wartości nNO po prowokacji alergenem wynikało najprawdopodobniej z narastającego obrzęku błony śluzowej jamy nosowej, skutkującego zamknięciem ujścia zatok przynosowych i tym samym utrudnieniem przepływu NO z zatok do jam nosowych [60]. Wang i wsp. badali stężenie nNO u dzieci w wieku 3-14 lat chorujących na przewlekły ANN. Wykazali, że wartości nosowego NO są wyższe u pacjentów z łagodnym ANN wg kryteriów ARIA w porównaniu ze zdrową grupą kontrolną. Najwyższe wartości NO obserwowano u dzieci chorujących na umiarkowany-ciężki ANN. Zarówno w grupie z łagodnym, jak i umiarkowanym-ciężkim ANN zaobserwowano spadek wartości nNO po miesiącu leczenia (mometazon i/lub lek antyhistaminowy) przy czym spadek był istotnie statystycznie większy w drugiej z badanych grup [61]. Podobnie Lee i wsp. zaobserwowali, że wartości nNO u pacjentów chorujących na ANN są wyższe niż w zdrowej grupie kontrolnej. Ponadto wykazano, że u pacjentów chorujących na przewlekły ANN, wartości nNO były istotnie statystycznie niższe, a FeNO wyższe w porównaniu z pacjentami z okresowym ANN [22].

Przedstawione powyżej dane sugerują, że pomiary nNO mogą być pomocne w diagnostyce i ocenie nasilenia stanu zapalnego w górnych drogach oddechowych, wskazując na jego eozynofilowe podłoże. Wartości nNO obniżają się w wyniku zastosowanego leczenia przeciwwzapalnego, co sugeruje możliwość ich wykorzystania w trakcie terapii ANN. Jednak użyteczność nNO tak w diagnostyce, jak i leczeniu pacjentów z ANN wymaga dalszych badań.

## Podsumowanie

Pomiary FeNO:

- są jedną ze skaładowych procesu diagnostycznegou pacjentów z objawami dotyczącymi dolnych dróg oddechowych, wskazując na obecność stanu zapalnego o podłożu eozynofilowym,
- znąjdują zastosowanie w monitorowaniu nasilenia stanu zapalnego w dolnych drogach oddechowych u pacjentów z astmą w trakcie leczenia, jak również u pacjentów z ANN,
- mogą by przydatne w diagnostyce niezytu nosa, wskazując na jego alergiczny charakter oraz wykazując związek z czasem trwania i przebiegiem ANN,
- mogą byc nieprawidłowe (zarowno podwyzszone, jaki obniżone) w wielu schorzeniach, nie tylko o podłożu alergicznym ale również w takich jak: mukowiscydoza, infekcje wirusowe dróg oddechowych, czy w przebiegu nieswoistych zapaleń jelit,
- istnieje wiele czynnikow wpływających na ich wynikm.in: uczulenie na niektóre alergeny, zwłaszcza całoroczne oraz warianty genetyczne NOS,
- wydają sie byc związane z zaburzeniami przemianylipidowej oraz występowaniem uczulenia na alergeny wziewne,

Z kolei pomiary nNO, choć nie są tak rozpowszechnione jak oskrzelowe, mogą dostarczyć istotnych informacji dotyczących obecności i nasilenia zapalenia eozynofilowego w obrębie górnych dróg oddechowych, stając się przydatnym narzędziem w opiece nad pacjentami z ANN.
